# Prevalence of and Factors Associated with Antibiotic Prescription in Gynecological Practices in Germany

**DOI:** 10.3390/antibiotics15010053

**Published:** 2026-01-04

**Authors:** Cleo Hieber, Karel Kostev, Marcel Konrad, Matthias Kalder

**Affiliations:** 1 Marburg University, Department of Gynecology and Obstetrics, University Hospital, 35043 Marburg, Germany; hieberc@students.uni-marburg.de (C.H.);; 2 Epidemiology, IQVIA, 60549 Frankfurt am Main, Germany; 3 Health & Social, FOM University of Applied Sciences for Economics and Management, 60486 Frankfurt am Main, Germany

**Keywords:** antibiotic prescribing, gynecology, outpatient care, women’s health, antimicrobial stewardship

## Abstract

**Background:** Antibiotics are commonly used in gynecology, yet only limited outpatient prescribing data are available in Germany. The aim of this study is to estimate the prevalence of antibiotic prescriptions in gynecological practices and to identify patient and diagnostic factors. **Methods:** A retrospective cross-sectional analysis was conducted using anonymized electronic records from the IQVIA Disease Analyzer, including 344,187 women aged ≥16 years who had at least one gynecological visit in 2024. The primary outcome of interest was the prescription of an antibiotic. Consequently, the prevalence of antibiotic prescriptions was calculated overall and stratified by age group. Associations between potential factors and antibiotic prescriptions were assessed using multivariable logistic regression. **Results:** The overall prescription prevalence was 8.4% (29,007/344,187). Regarding the age distribution within the prescribed sample, the highest percentages were observed among women aged 31–40 years (25.6%) and 16–30 years (25.4%), while those aged 51–60 and >60 made up 12.9% and 19.1%, respectively. The most commonly prescribed agents were fosfomycin trometamol (35.9%), clindamycin (17.6%), and pivmecillinam (10.7%). Mastitis (OR 63.54, 95% CI 55.79–72.38), acute cystitis (OR 43.67, 95% CI 41.63–45.80), and unspecified urinary tract infection (OR 31.58, 95% CI 20.11–33.12) were strongly positively associated with AB prescription. Positive associations were also observed for acute vaginitis (OR 3.44, 95% CI 3.30–3.58), chlamydial infection (OR 6.27, 95% CI 5.77–6.81), and pregnancy (OR 1.95, 95% CI 1.85–2.05). Negative associations were observed for dysmenorrhea (OR 0.52, 95% CI 0.48–0.56), irregular menstruation (OR 0.65, 95% CI 0.60–0.71), menopausal disorders (OR 0.51, 95% CI 0.48–0.53), and ovarian cysts (OR 0.78, 95% CI 0.72–0.84). **Conclusions:** Antibiotic use in gynecology is low and strongly diagnosis-driven, primarily for urogenital infections. Signals of inappropriate prescribing in patients with candidiasis suggest a need for improved diagnostic accuracy and guideline adherence.

## 1. Introduction

The prescription of antibiotics constitutes an indispensable component of modern medical practice, including gynecology [1,2]. In this field, antibiotics are essential for preventing and treating infections such as urinary tract infections (UTIs), bacterial vaginosis, and pregnancy-related complications [2,3,4]. Approximately 71% of UTIs are managed with antibiotic therapy [4]. Within the field of German obstetrics, the majority of prescriptions are issued in the context of cesarean sections (64.3%) and premature rupture of membranes (41.2%), with antibiotic exposure documented in 64.4% of pregnant women during pregnancy or labor [5]. European studies suggest that factors such as maternal age, gestational age, body mass index, smoking status, and chronic illnesses can influence antibiotic exposure during pregnancy [5,6].

However, prescribed therapies are not always appropriate, which is concerning given the escalating global problem of antimicrobial resistance [1,3,7]. Resistance increases the risk of treatment failure, recurrent infections, and disruption to the maternal microbiome. This, in turn, has potential consequences for the neonatal microbiome [8,9].

While Schilling et al. provided initial insights into factors influencing antibiotic exposure during pregnancy in Germany, their single-center study with its small cohort has limited generalizability. Regional differences could not be considered, and there is a lack of data concerning broader populations. Beyond pregnancy, there is a notable absence of comprehensive studies investigating factors associated with antibiotic therapy in gynecological practices. Indeed, even fundamental research on appropriate therapeutic approaches is scarce for certain diagnoses. In light of increasing antimicrobial resistance, a nationwide analysis is clearly urgently needed.

This study aimed to analyze the prevalence of antibiotic prescriptions in gynecological practices and to investigate the association of patient age and specific diagnoses with prescribing patterns.

## 2. Results

### 2.1. Prevalence of Antibiotic Prescription

**Figure 1:**
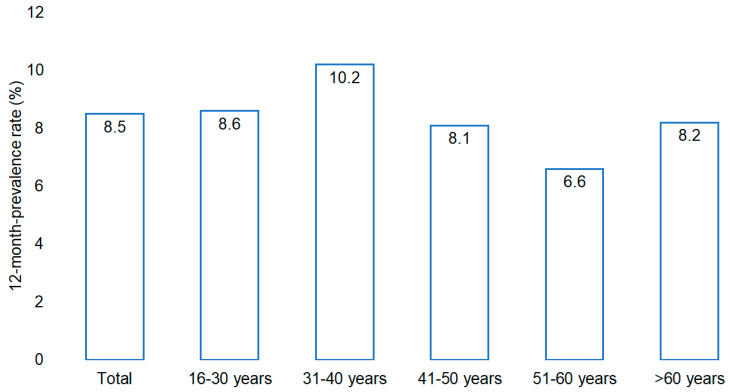
Average prevalence of antibiotic prescriptions in women treated by 220 gynecologists in Germany in 2024.

In 2024, the overall prevalence of at least one systemic antibiotic prescription issued in outpatient gynecological care among women treated by the 220 gynecologists included in the Disease Analyzer database was 8.4% (29,007/344,187 patients). As illustrated in Figure 1, prevalence differed across age groups. The highest prescription rates were observed among women aged 31–40 years and those aged 16–30 years, while lower rates were noted in the group of women aged 51–60 years. Among women over 60 years of age, the prevalence of antibiotic prescriptions was moderate.

**Figure 2:**
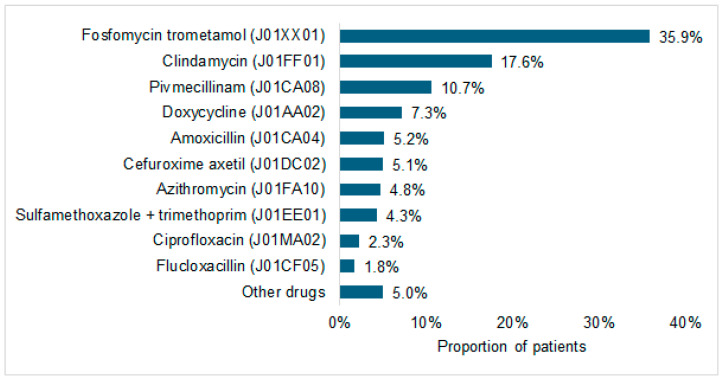
Proportion of patients receiving different systemic antibiotic agents at the index prescription in outpatient gynecological care in 2024.

The composition of antibiotic prescriptions is illustrated in Figure 2. Fosfomycin trometamol accounted for the largest share (35.9%) of all prescriptions, followed by clindamycin (17.6%) and pivmecillinam (10.7%). Other drugs were prescribed less frequently.

### 2.2. Basic Characteristics of the Study Sample

The baseline characteristics of the study population are summarized in Table 1. On average, women who received antibiotics (n = 29,007) were slightly younger than those who did not (43.8 ± 17.3 vs. 45.2 ± 17.1 years, *p* < 0.001). Given that this narrow difference is of limited clinical significance, the age stratification presented below offers greater clinical insight. The age distribution also differed significantly between groups. Women aged 31–40 years accounted for a larger share of the antibiotic group compared to the control group (25.6% vs. 20.8%, *p* < 0.001), whereas the proportion of women aged 51–60 and >60 years was lower among those prescribed antibiotics.

Marked differences were observed in gynecological and urogenital diagnoses. Acute infections, including acute cystitis (23.0% vs. 1.0%), unspecified urinary tract infection (19.5% vs. 1.1%), acute vaginitis (16.8% vs. 6.2%), and mastitis (3.3% vs. 0.1%) (all *p* < 0.001), were strongly overrepresented among women with antibiotic prescriptions. Several chronic or recurrent conditions also differed between the groups. Chlamydial infection (3.8% vs. 0.8%), candidiasis of the vulva and vagina (6.2% vs. 3.1%), and pregnancy-related diagnoses (11.1% vs. 5.2%) were more prevalent among women prescribed antibiotics. By contrast, menopausal and perimenopausal disorders (9.5% vs. 16.6%), dysmenorrhea (2.9% vs. 5.0%), and irregular menstruation (2.9% vs. 4.6%) were more prevalent among women not prescribed antibiotics.

### 2.3. Association of Age and Pre-Defined Disorders with Antibiotic Prescriptions

Multivariable logistic regression analysis revealed that acute infectious diagnoses were strongly associated with antibiotic prescription (Table 2). Mastitis showed the strongest association: Women with this diagnosis were more than 60 times more likely to receive antibiotics (OR 63.54, 95% CI 55.79–72.38, *p* < 0.001). Similarly, acute cystitis (OR 43.67, 95% CI 41.63–45.80, *p* < 0.001) and unspecified urinary tract infection (OR 31.58, 95% CI 20.11–33.12, *p* < 0.001) were very strongly associated with antibiotic use. Acute vaginitis also significantly increased the likelihood of prescription (OR 3.44, 95% CI 3.30–3.58, *p* < 0.001).

Among chronic or recurrent conditions, chlamydial infection (OR 6.27, 95% CI 5.77–6.81, *p* < 0.001) and pregnancy-related diagnoses (OR 1.95, 95% CI 1.85–2.05, *p* < 0.001) were associated with higher levels of antibiotic prescription, whereas candidiasis of the vulva and vagina showed only a moderate positive association (OR 1.16, 95% CI 1.09–1.24, *p* < 0.001).

Conversely, several gynecological conditions were negatively associated with antibiotic prescriptions. Women with dysmenorrhea (OR 0.52, 95% CI 0.48–0.56), irregular menstruation (OR 0.65, 95% CI 0.60–0.71), and menopausal or perimenopausal disorders (OR 0.51, 95% CI 0.48–0.53) were significantly less likely to receive antibiotics (all *p* < 0.001). Unspecified ovarian cysts were also inversely related to prescriptions (OR 0.78, 95% CI 0.72–0.84, *p* < 0.001).

Age showed only moderate associations. Compared with women aged 16–30 years, women aged 31–40 years (OR 1.16, 95% CI 1.12–1.21) and women >60 years (OR 1.06, 95% CI 1.02–1.12) were slightly more likely to receive antibiotics, whereas women aged 51–60 years were marginally less likely to receive antibiotics (OR 0.95, 95% CI 0.90–1.01, *p* = 0.042).

## 3. Discussion

This nationwide study revealed an overall antibiotic prescription prevalence of 8.4% in gynecological practices, with fosfomycin trometamol and clindamycin accounting for the largest shares. Prescription patterns varied significantly by age and were most strongly driven by acute infectious diagnoses such as mastitis and cystitis. Prescription rates varied across age groups and were strongly associated with specific diagnoses. To our knowledge, no previous work has comprehensively examined antibiotic prescribing in outpatient gynecology. It should be emphasized that this study does not assess the clinical indication for which antibiotics were prescribed; rather, it examines associations between documented diagnoses within predefined time windows and the likelihood of receiving an antibiotic prescription.

To our knowledge, this is the first nationwide study in Germany to examine antibiotic prescribing in outpatient gynecological care using routine data, providing diagnosis-specific insights into real-world prescribing patterns.

Our findings can also be interpreted in the context of national surveillance data reported to the WHO Global Antimicrobial Resistance and Use Surveillance System (GLASS). While Germany contributes reimbursement and dispensing data that can be disaggregated by age and gender, these surveillance metrics often lack specific clinical context regarding the indication for treatment. Our study complements this surveillance by providing the missing link between consumption and indication. Having this information is key to understanding surveillance trends and to designing better, more targeted stewardship programs [10].

A German cross-sectional study using AOK Nordost data from 2009 reported that gynecologists accounted for a median of 1.5% of outpatient antibiotic prescriptions [11]. Although Zweigner et al. focused on the contribution to overall consumption rather than patient-level prescription rates, our findings align with their observation: antibiotics are used sparingly in gynecology, with more than 90% of patients not receiving a prescription. While maintaining low prescription rates is crucial for preventing resistance and side effects [1,8,9], it remains essential that patients with bacterial infections receive appropriate therapy. The absence of prescriptions in same cases with infection diagnoses may reflect conservative management of mild or non-bacterial conditions.

Age had only a moderate influence, with slightly higher prescription levels observed among women aged 31–40, 41–50, and >60 years, while those aged 51–60 were less likely to receive antibiotics. The highest prevalence was observed in women aged 31–40, followed by those aged 16–30, which is consistent with prior German studies [4,12]. The higher exposure rate among younger women likely reflects sexually transmitted infections (STIs) and routine chlamydia screening up to the age of 25 [13,14,15,16]. Additionally, pregnancy contributes to higher prescription rates in these age groups, as infections during pregnancy often require treatment to prevent complications [5].

Mastitis showed the strongest association with antibiotic prescription in this study (OR 63.54), indicating that this diagnosis was documented substantially more often among women who received an antibiotic prescription than among those who did not. This is consistent with international data showing high prescribing rates worldwide [17]. Current guidelines recommend cephalosporins or β-lactamase inhibitor–protected penicillins for mastitis [18]. While these agents were not among the most frequently prescribed antibiotics overall in our study, indication-specific prescribing could not be assessed.

Urinary tract infections were the most common indication, with acute cystitis (OR 43.67) and unspecified UTI (OR 31.58) being strongly associated with prescribing. This aligns with prior German data reporting antibiotic treatment rates of ~70% for UTIs [4]. Fosfomycin trometamol was the most commonly prescribed agent, which is consistent with guideline recommendations [19,20]. Pivmecillinam was also frequently used, reflecting evolving practice patterns following restrictions on fluoroquinolone [21,22].

Chlamydial infection was strongly associated with antibiotic therapy (OR 6.27), in line with international data [14]. Current German guidelines recommend doxycycline or ceftriaxone for non-pregnant patients, and azithromycin for pregnant women [23]. In our study, doxycycline was among the most commonly prescribed agents, likely due to its use in treating chlamydia.

Pregnancy was associated with an increased rate of prescription (OR 1.95), which is consistent with prevalence estimates of 15–25% in Germany [12,24], and with higher rates observed in France and Sweden [6,24]. This increased rate is linked to pregnancy-related infections and physiological changes that predispose patients to UTIs [25]. Screening programs also contribute to the higher level of prescriptions in this cohort [26]. Inpatient studies report higher rates, largely due to prophylactic use during childbirth [5,27].

One notable finding is the positive association between candidiasis and antibiotic prescriptions (OR 1.16). It should be emphasized that this analysis does not assess the indication for which antibiotics were prescribed; diagnoses of candidiasis reflect documentation within the predefined time window and do not imply that antibiotics were used to treat fungal infections. While this could reflect inappropriate prescribing, given that guidelines recommend antifungal therapy only [28], we cannot rule out that these prescriptions were intended for concurrent bacterial infections (e.g., bacterial vaginosis or UTI). Regardless of the indication, this association warrants caution, as antibiotics can exacerbate vulvovaginal candidiasis by disrupting the microbiome [8,28], highlighting the need for accurate differential diagnosis.

As expected, chronic or recurrent gynecological conditions such as dysmenorrhea, irregular menstruation, and menopausal disorders were negatively associated with antibiotic prescribing. This inverse association likely reflects the composition of the study population: women presenting with these non-infectious conditions constitute a large subgroup seeking care for functional complains. As the comparison group includes patients with acute infections, these diagnoses appear statistically associated with a lower prescription rate relative to the overall study population. These conditions are typically managed with NSAIDs, hormone therapy, psychotherapy, or surgical interventions [29,30,31].

The strengths of this study include its large, representative sample and the use of routine practice data, which minimizes recall bias. Limitations include a lack of information on disease severity, resistance profiles, and laboratory results, as well as potential coding inconsistencies. Since the dataset records issued prescriptions rather than dispensed medication, primary non-adherence is not captured, likely resulting in an overstimulation of actual consumption. Furthermore, the spectrum of analyzed covariates was restricted to available database fields, excluding other potential sociodemographic factors. Prescribing behavior may also cluster within practices, limiting the independence of observations.

## 4. Methods

### 4.1. Database

This investigation was based on anonymized electronic medical records retrieved from the IQVIA™ Disease Analyzer database, which contains patient demographic information (e.g., age, sex), medical diagnoses, and prescriptions. These data are obtained directly from the practice software used by office-based general practitioners and specialists in Germany [32]. In 2024, the database encompassed approximately 3000 physicians across multiple specialties, including 220 gynecologists. These were selected using a stratified panel design based on specialty group, federal state, community size, and physician age. Previous evaluations have demonstrated that the database is representative of outpatient care in Germany [32], and it has been widely applied in studies on women’s health [33,34,35]. All gynecological practices that actively contributed data to the Disease Analyzer database in 2024 were included in the present analysis (n = 220); no additional sampling was performed.

### 4.2. Study Population

This retrospective cross-sectional study included 344,187 women aged ≥16 years who had at least one consultation with a participating gynecologist in 2024. All women aged ≥16 years who had at least one consultation with a participating gynecologist in 2024 were included in the study population. Antibiotic prescription status during the year was used for descriptive and analytical purposes but did not define inclusion into the study.

For women who received an antibiotic prescription, the date of the first prescription in 2024 was defined as the index date to serve as a temporal reference for assessing diagnoses in relation to the prescription. For women without an antibiotic prescription, a randomly selected consultation date in 2024 was assigned as the index date to allow comparable assessment of diagnoses within predefined time windows.

### 4.3. Study Outcomes

The primary outcome of interest was the issuance of at least one systemic antibiotic prescription to a patient during the observation year 2024. Antibiotic prescriptions were identified using the World Health Organization Anatomical Therapeutic Chemical (ATC) classification system and included all systemic antibacterial agents (ATC code J01) prescribed in outpatient gynecological care.

Secondary outcomes included the overall prevalence of antibiotic prescribing among women treated in gynecological practices, as well as age-specific prescription prevalence stratified into the following groups: 16–30, 31–40, 41–50, 51–60, and >60 years. In addition, the distribution of prescribed antibiotic agents was analyzed to describe prevailing prescribing patterns.

Furthermore, factors associated with antibiotic prescription were examined, including patient age and predefined acute and chronic or recurrent gynecological or urogenital diagnoses. Acute diagnoses were assessed within seven days prior to or on the index date, while chronic or recurrent diagnoses were assessed within six months prior to or on the index date, as described in detail below.

The selected time windows for diagnostic assessment were based on established clinical documentation practices rather than formal ICD-10 temporal definitions. Acute conditions (e.g., cystitis, vaginitis, mastitis) were assessed within a seven-day window before or on the index date to capture diagnoses directly related to the antibiotic prescription decision, reflecting their short clinical course and typical coding at or near the time of treatment. In contrast, chronic or recurrent conditions (e.g., menstrual disorders, menopausal disorders, pregnancy) were assessed within a six-month window to account for their longer duration and the fact that such diagnoses are often documented intermittently rather than at every visit. This approach has been applied in prior analyses using the Disease Analyzer database to ensure clinically meaningful attribution of diagnoses to prescribing behavior. The complete set of diagnostic codes is presented in Table 3.

### 4.4. Variables and Statistical Analyses

The prevalence of antibiotic prescription was calculated as the proportion of women who received at least one systemic antibiotic prescription during the calendar year 2024 among all women treated in gynecological practices in that year. Age-specific prevalence rates were calculated by dividing the number of patients with an antibiotic prescription in a specific age group by the total number of patients in that age group. For descriptive comparisons between women with and without antibiotic prescriptions, categorical variables were compared using χ^2^ tests and continuous variables using *t*-tests. These analyses were performed for descriptive purposes only.

A multivariable logistic regression model was applied to identify factors associated with AB use. The dependent variable was antibiotic prescription (yes/no), and the independent variables were age group and the predefined diagnoses. All variables included in the multivariable models were selected a priori based on clinical relevance and data availability. Bivariable analyses were not used for variable selection, as the aim was to estimate adjusted associations while accounting for potential confounding rather than to develop a parsimonious predictive model.

Odds ratios (ORs) and 95% confidence intervals (CIs) were reported. A two-sided *p* < 0.05 was considered statistically significant. Given the very large sample size of the study, formal goodness-of-fit statistics for the logistic regression model (e.g., Hosmer–Lemeshow test) were not emphasized, as such tests may indicate statistically significant lack of fit even in the presence of clinically negligible deviations. Accordingly, the analysis focused on reporting adjusted effect estimates (odds ratios with 95% confidence intervals), which are most relevant to the study objective. All analyses were performed using SAS version 9.4 (SAS Institute, Cary, NC, USA).

## 5. Conclusions

In conclusion, this study provides the first comprehensive overview of antibiotic prescribing in the field of outpatient gynecology in Germany. The results demonstrate a generally conservative prescribing behavior with an overall prevalence of 8.4%, largely dominated by the treatment of acute cystitis using fosfomycin. These findings underscore the importance of ongoing surveillance and guideline adherence, particularly to bridge the research gap between the extensive literature on UTIs and pregnancy and the limited evidence regarding antibiotic therapy for other gynecological conditions.

## Figures and Tables

**Table 1:** Age and diagnosis data of study patients.

Variable	Proportion Among Women with Antibiotic Prescription (N, %)	Proportion Among Women Without Antibiotic Prescription (N, %)	*p*-Value
Number of patients	29,007	315,180	
Age (mean, SD)	43.8 (17.3)	45.2 (17.1)	<0.001
Age 16–30 years	7365 (25.4)	78,692 (25.0)	<0.001
Age 31–40 years	7424 (25.6)	65,671 (20.8)	
Age 41–50 years	4928 (17.0)	55,666 (17.7)	
Age 51–60 years	3737 (12.9)	52,948 (16.8)	
Age > 60 years	5553 (19.1)	62,203 (19.7)	
Acute vaginitis	4882 (16.8)	19,471 (6.2)	<0.001
Acute cystitis	6673 (23.0)	3047 (1.0)	<0.001
Urinary tract infection, site not specified	5644 (19.5)	3446 (1.1)	<0.001
Mastitis without abscess	946 (3.3)	317 (0.1)	<0.001
Chlamydial infection, unspecified	1092 (3.8)	2556 (0.8)	<0.001
Candidiasis of vulva and vagina	1787 (6.2)	9822 (3.1)	<0.001
Unspecified ovarian cysts	1304 (4.5)	12,796 (4.1)	<0.001
Irregular menstruation, unspecified	852 (2.9)	14,579 (4.6)	<0.001
Dysmenorrhea, unspecified	846 (2.9)	15,767 (5.0)	<0.001
Menopausal and perimenopausal disorders	2740 (9.5)	52,246 (16.6)	<0.001
Pregnancy	3225 (11.1)	16,399 (5.2)	<0.001

**Table 2:** Association between age/predefined diagnoses and antibiotic prescription in patients followed by gynecologists in Germany.

Variable	OR (95% CI) *	*p*-Value
Age 16–30 years	Reference	
Age 31–40 years	1.16 (1.12–1.21)	<0.001
Age 41–50 years	1.05 (1.01–1.10)	0.035
Age 51–60 years	0.95 (0.90–1.01)	0.042
Age > 60 years	1.06 (1.02–1.12)	<0.001
Acute vaginitis	3.44 (3.30–3.58)	<0.001
Acute cystitis	43.67 (41.63–45.80)	<0.001
Urinary tract infection, site not specified	31.58 (20.11–33.12)	<0.001
Mastitis without abscess	63.54 (55.79–72.38)	<0.001
Chlamydial infection, unspecified	6.27 (5.77–6.81)	<0.001
Candidiasis of vulva and vagina	1.16 (1.09–1.24)	<0.001
Unspecified ovarian cysts	0.78 (0.72–0.84)	<0.001
Irregular menstruation, unspecified	0.65 (0.60–0.71)	<0.001
Dysmenorrhea, unspecified	0.52 (0.48–0.56)	<0.001
Menopausal and perimenopausal disorders	0.51 (0.48–0.53)	<0.001
Pregnancy	1.95 (1.85–2.05)	<0.001

* Multivariate logistic regression adjusted for age and for all diagnoses listed in the table; *p* < 0.05 is considered statistically significant.

**Table 3:** Diagnosis codes used in the study.

Diagnosis	ICD-10 Code
Diagnosis documented within seven days prior to or on index date	
Acute vaginitis	N76.0
Acute cystitis	N30.0
Urinary tract infection, site not specified	N39.0
Mastitis without abscess	N61.0
Diagnosis documented within six months prior to or on index date	
Chlamydial infection, unspecified	A74.9
Candidiasis of vulva and vagina	B37.3
Unspecified ovarian cysts	N83.2
Irregular menstruation, unspecified	N92.6
Dysmenorrhea, unspecified	N94.6
Menopausal and perimenopausal disorders	N95
Pregnancy	Z32.1, Z33, Z34, Z35

## Data Availability

Data were obtained from IQVIA and are available upon reasonable request with the permission of IQVIA. Restrictions apply due to data protection requirements.
